# Pre-Natal Influences as Affecting the Teeth

**Published:** 1883-12

**Authors:** Geo. H. Cushing

**Affiliations:** Professor of Operative Dentistry in the Chicago Dental Infirmary


### ARTICLE III.

PRE-NATAL INFLUENCES AS AFFECTING THE TEETH. By GEO. H. CUSHING, D.D.S., Professor of Operative Dentistry in the Chicago Dental Infirmary.

In considering the pre-natal conditions that may influence the character of the teeth, we must start with the proposition that perfect and healthy children can only spring from perfect and healthy parents.

While this implies that just so far as the parents diverge from the conditions of physical perfection and health, just so far will the offspring be physically imperfect and unhealthy, yet it will hereafter be seen that this proposition must to some extent be qualified.

Considering man simply in the nature of an animal, it would seem not unreasonable to suppose that if he were living in a purely natural state he would be almost or quite free from defective teeth.

So far as we know, the wild animals are, as a rule, exempt from diseases of the teeth, and they reproduce their kind with close approach to perfection of type; and, were the animal *man* under like natural conditions, the same results ought to be predicted of him.

But whether the conclusions naturally to be drawn from the last proposition, viz., that decay of the teeth is a disease due to civilization, be accepted or not, it probably will not be denied that man is living, in a certain sense, in an unnatural condition, and that the state which we term civilization tends more and more to force him into artificial conditions of life, farther and farther removed from the simple methods intended by nature for his existence, and that this must tend in a greater or less degree to modify his physical status.

So we are forced to consider this question from the standpoint of the present status of man with reference to his perfection or otherwise as an animal, and his healthy and unhealthy condition.

Under what conditions, then, do we as a general rule see the reproduction of the species conducted? In no case where either parent is in a perfect physical condition, and often with one or both parents in an absolutely unfit condition to become progenitors because of their being in a pronouncedly unhealthy condition.

Parents in the last stages of consumption, with incurable diseases of many kinds, with scrofulous diatheses, and those with constitutions utterly wrecked by over-indulgence and long-continued violation of the laws of health, or by reason of repeated attacks of various acute diseases, are continually and always bringing children into the world.

What can be expected of the offspring of such parents? Only that their organizations will be to a greater or less extent imperfect even under the most careful attention to the conditions of the mother during the period of gestation and lactation.

In so brief a paper as this, it would be impossible to go to any extent into the consideration of all the pre-natal influences which might modify the condition of offspring. The laws of heredity under which certain types may be handed down from remote ancestors; the accidents and acute diseases to which the most healthy women are liable during pregnancy ; and the seeming contradictions of physiological laws in well-known observed cases, all these things have a marked influence, but they can be only incidentally alluded to.

Under the general proposition that only healthy parents can beget healthy offspring, it becomes apparent that the utmost care and attention should be given toward perfecting the health of the mother from the moment of conception till she ceases to nourish her child.

This care of course secures the highest possible development of the child in all respects, but its importance as regards the teeth is greater than in any other direction, for the reason that the teeth retain with comparatively little modification, the character which is given them at the time of their formation. The other parts of the body may have been but imperfectly nourished and so but imperfectly formed and developed and yet the recuperative power of the constitution may subsequently bring up to a near approach to health that which was imperfectly begotten, while the teeth will always bear testimony to their imperfect original construction. Consequently everything that tends to promote the health of the mother is indispensable to the perfection of the teeth, while everythnig calculated in any degree to lower the standard of health in the mother will be sure to prove more or less injurious to the teeth of the child.

It would be a work of supererogation to enumerate here the course and methods that mothers should pursue to secure the highest physical condition,—the whole matter would be summed up by saying she should conform entirely to hygienic laws, but it will never be out of place to emphasize the importance of mothers giving extraordinary attention to this subject.

You need only to cast your glance upon an hundred mothers whom you have known during the period of the rearing of their young, to note how entirely indifferent they generally seem to be with regard to this matter. The lives of excitement they lead, the late hours they keep, the pernicious habit of tight-lacing that many of them follow, their neglect of exercise and the kind of food they eat, all tend to show their indifference, while the persistence in such habits can tend to but one result, viz.: the bringing into life of very imperfectly formed and poorly nourished offspring.

In a general sense there can be no controversy with regard to the propositions thus far so briefly laid down, and yet we see occasionally seeming contradictions of them. We may sometimes see parents of very poor physical condition, and whose teeth are of the poorest quality whose children will prove to be remarkably healthy and their teeth will astonish us by their strength and immunity from decay (caries). In such cases the law of heredity may and doubtless does play an important part, and the child probably inherits from remote ancestors the fine physique and strong teeth which belonged to neither of his immediate ancestors.

On the other hand, we may see the very reverse of this in the case of parents who are in comparatively perfect physical condition, with strong and sound teeth whose children will have an imperfect physique and very frail teeth. The same law of heredity may also explain this anomaly.

Again, we may find that a mother who will be in miserable health during the entire period of utero-gestation, whose appetite will be poor and capricious, and who does not take sufficient or properly nourishing food, may bring forth the most robust and healthy child, while on the other hand a mother who may pass through this entire period seemingly in perfect health, and who may strictly observe hygienic laws, will produce a delicate and sickly child. These last cited cases are in seeming contradiction to physiological laws, but they, with the others referred to, are only exceptions to the general rule and must not be counted on as any excuse for the slightest relaxation of vigilance over the health of all mothers during the period of gestation and lactation.

Of the influence of food upon the teeth, it might be thought that what has already been said would cover the whole ground, but it does not fully do so. Of course, if we had healthy parents to deal with, and they understood the laws of hygiene, and appreciated the importance of the strictest compliance with those laws, there would be no necessity for saying anything more. But, unfortunately, we have not such people to deal with generally; and sometimes those who have an understanding of what is proper, and who seek to do all in their power to secure the highest physical condition for themselves, and consequently for their offspring, are unable, from diseased or morbid conditions, to obey every hygienic requirement. Many mothers, during the period of child-bearing, are very capricious in their appetites, often craving food that is the least desirable for them on general principles, and loathing that which would most certainly supply the requisite elements for their own and their child's complete nourishment. In such cases we must attempt to supply, by artificial means or by specially prepared foods, the elements which fail to be assimilated in the ordinary way.

As affecting the teeth especially, though also the general system the lack of the phosphate of lime in the tissues, is particularly observed in cases like those just referred to. These phosphates should be furnished in the ordinary diet in ample proportions; but in some cases, as above stated, the mother can not, and in others *will* not, use the proper diet; while in other cases, in which the most desirable food is taken, and, although digestion seems to be perfectly performed, yet the phosphates are evidently not thoroughly assimilated.

In many cases where the phosphates fail to be assimilated from the natural food they do seem to be from other sources.

It has been claimed by some practitioners that they have succeeded in materially improving the conditions of the teeth of offspring by the administration to mothers during pregnancy of the crude phosphate of lime.

Later, the syrup of the lacto phosphate of lime was introduced and extraordinary claims were made for it by the French physician who first called attention to it. He claimed that by the preparation of the phosphate of lime with lactic acid it was partially digested before being taken into the stomach, and thus was more easily and surely assimilated. Many practitioners, both of dentistry and medicine, feel confident that they have seen excellent results follow its use.

Another form of phosphates which greatly commends itself, is the preparation of wheat phosphates made by E. H. Sargent & Co., of Chicago. This seems to furnish an admirable method of administering the required elements, and is perhaps preferable to any other. It is simply a condensed and easily digested form of phosphatic food, though there may be cases in which the syrup of the lacto phosphate of lime would be more easily tolerated.

There can be no question but that the habitual use of Sargent's preparation or of the syrup, by mothers during pregnancy and lactation, is likely to prove beneficial to the teeth of the children, and also to those of the mothers, and it should always be prescribed where there is probability of imperfection in the dental organs from either inheritance or impaired health of the mother.

The administration of either should be intermittent—that is, it should be used, say for a month, and abandoned for a month, then resumed and again abandoned, and so on.

Imperfectly as this subject has been presented, it should arouse the attention of the profession to the importance of urging upon all our patients, who are about to become mothers, the great necessity and the duty of their using every means within their reach that is likely to tend toward improving the teeth of their offspring, and to this end dentists should ask the coöperation of the family physician, and of the husband, which will be readily and gladly accorded when once its importance is apparent to them.

The younger members of the profession should give this subject their serious consideration, for the amount of good they may accomplish by making one of the cardinal principles of their practice the urging of this pre-natal care upon their patients can never be fully estimated, while the laurels they may win in establishing successful prophylactic treatment will be those they may well be proud to wear.

---

STATEMENT OF MORTALITY FROM BUREAU OF VITAL STATISTICS FOR SEPTEMBER gives the monthly death rate as 1.73 per 1,000. Total deaths, 970. Total deaths in August, 1,163. September, 1882, 1,032. September, 1881, 1,211. Greatest number of deaths from diarrhœal diseases, 143; diphtheria, 45; scarlatina, 36; typhoid fever, 44; croup, 17; typho malarial, 12; phthisis pulmonalis, 94; acute lung diseases, 72; pertussis, 12; cerebral meningitis, 23; Bright's disease, 9; inanition, 22.

